# Plasma and synovial fluid microRNAs as potential biomarkers of rheumatoid arthritis and osteoarthritis

**DOI:** 10.1186/ar3013

**Published:** 2010-05-14

**Authors:** Koichi Murata, Hiroyuki Yoshitomi, Shimei Tanida, Masahiro Ishikawa, Kohei Nishitani, Hiromu Ito, Takashi Nakamura

**Affiliations:** 1 Department of Orthopaedic Surgery, Kyoto University Graduate School of Medicine, 54 Kawahara-cho, Shogoin, Sakyo, Kyoto 606-8507, Japan

## Abstract

**Introduction:**

MicroRNAs (miRNAs), endogenous small noncoding RNAs regulating the activities of target mRNAs and cellular processes, are present in human plasma in a stable form. In this study, we investigated whether miRNAs are also stably present in synovial fluids and whether plasma and synovial fluid miRNAs could be biomarkers of rheumatoid arthritis (RA) and osteoarthritis (OA).

**Methods:**

We measured concentrations of miR-16, miR-132, miR-146a, miR-155 and miR-223 in synovial fluid from patients with RA and OA, and those in plasma from RA, OA and healthy controls (HCs) by quantitative reverse transcription-polymerase chain reaction. Furthermore, miRNAs in the conditioned medium of synovial tissues, monolayer fibroblast-like synoviocytes, and mononuclear cells were examined. Correlations between miRNAs and biomarkers or disease activities of RA were statistically examined.

**Results:**

Synovial fluid miRNAs were present and as stable as plasma miRNAs for storage at -20°C and freeze-thawing from -20°C to 4°C. In RA and OA, synovial fluid concentrations of miR-16, miR-132, miR-146a, and miR-223 were significantly lower than their plasma concentrations, and there were no correlation between plasma and synovial fluid miRNAs. Interestingly, synovial tissues, fibroblast-like synoviocytes, and mononuclear cells secreted miRNAs in distinct patterns. The expression patterns of miRNAs in synovial fluid of OA were similar to miRNAs secreted by synovial tissues. Synovial fluid miRNAs of RA were likely to originate from synovial tissues and infiltrating cells. Plasma miR-132 of HC was significantly higher than that of RA or OA with high diagnosability. Synovial fluid concentrations of miR-16, miR-146a miR-155 and miR-223 of RA were significantly higher than those of OA. Plasma miRNAs or ratio of synovial fluid miRNAs to plasma miRNAs, including miR-16 and miR-146a, significantly correlated with tender joint counts and 28-joint Disease Activity Score.

**Conclusions:**

Plasma miRNAs had distinct patterns from synovial fluid miRNAs, which appeared to originate from synovial tissue. Plasma miR-132 well differentiated HCs from patients with RA or OA, while synovial fluid miRNAs differentiated RA and OA. Furthermore, plasma miRNAs correlated with the disease activities of RA. Thus, synovial fluid and plasma miRNAs have potential as diagnostic biomarkers for RA and OA and as a tool for the analysis of their pathogenesis.

## Introduction

MicroRNAs (miRNAs) are endogenous small (approximately 22 nucleotides) noncoding RNAs and regulate the activities of target mRNAs by binding at sites in the 3' untranslated region of the mRNAs [1,2], and currently more than 721 human miRNAs have been registered [3]. miRNAs have been implicated in important cellular processes such as lipid metabolism [4], apoptosis [5], differentiation [6], organ development [7] and malignant tumors [8-12], and there is a prediction that one-third of all mRNAs may be regulated by miRNAs [13]. Recently Mitchell *et al *showed that miRNAs are present in human plasma in a remarkably stable form that is protected from endogenous RNase activity [14]. Furthermore, miRNAs are present in dried biological fluids such as semen, saliva, vaginal secretions, and menstrual blood [15], and expected to be diagnostic and prognostic biomarkers of various cancers [14,16,17].

Several cellular or tissue miRNAs associate with rheumatoid arthritis (RA). The expressions of miR-155, miR-146a, and miR-124a in RA fibroblast-like synoviocytes (FLSs); miR-146 and miR-155 in RA synovial tissue; or miR-146a, miR-155, miR-132, and miR-16 in RA peripheral blood (PB) mononuclear cells (MNCs) are upregulated compared with osteoarthritis (OA) or healthy controls (HCs) [18-21].

On the other hand, there is no report associated with miRNAs in plasma or synovial fluid of RA or OA patients. In this study, we investigated the presence and the stability of miRNAs in synovial fluid, and compared synovial fluid miRNAs with plasma miRNAs. We also examined the differences in the expression of plasma miRNAs or in synovial fluid miRNAs between RA, OA and HC, and the correlation of plasma or synovial fluid miRNAs with disease activities of RA.

## Materials and methods

### Preparation of blood and joint fluid samples

Ethical approval for this study was granted by the ethics committee of Kyoto University Graduate School and Faculty of Medicine. Informed consent was obtained from 108 participants (40 with RA, 38 with knee OA, and 30 as HC, Tables 1 and 2). According to the request of the ethics committee, HCs were limited between 20 and 65 years old. RA and OA were diagnosed according to the criteria of the American College of Rheumatology [22,23]. Both peripheral blood and synovial fluid were obtained from 20 patients with RA and 22 patients with OA. Blood samples were collected with ethylenediaminetetraacetic acid dipotassium salt (EDTA-2K) containing tube to separate plasma. Both of samples were centrifuged 400 g for seven minutes and stored at -20°C until analyses.

**Table 1:** Clinical features of the participants who contributed plasma

Characteristics	RA	OA	HC
Number of participants	30	30	30
Sex, male/female	8/22	7/23	13/17
Age, mean (range)	60.1 (22 to 77)	75.1 (65 to 89)	46.5 (32 to 62)
Disease duration (y), mean (range)	10.4 (0.3 to 32)	NA	NA
Positive anti-CCP antibody, n (%)	10 (90.9%)^†^	NA	NA
ESR (mm), mean (range)	37.2 (4 to 116)	NA	NA
CRP (mg/dl), mean (range)	2.1 (0 to 9.6)	NA	NA
MMP3 (ng/ml), mean (range)	290.1 (32.4 to 800)	NA	NA
DAS28, mean (range)	4.4 (1.7 to 7.1)	NA	NA
SJC, mean (range)	4.3 (0 to 13)	NA	NA
TJC, mean (range)	4.5 (0 to 27)	NA	NA
VAS	42.3 (0 to 95)	NA	NA
Steinbrocker Stage, n	I: 4, II: 3, III: 6, IV: 17	NA	NA
Steinbrocker Class, n	I: 1, II: 24, III: 5, IV: 0	NA	NA
Kellgren/Lawrence grade, n	NA	I: 0, II: 0, III: 9, IV: 21	NA
Medication, n (%)			
Prednisolone	21 (70%)	NA	NA
Methotrexate	18 (60%)	NA	NA
Infliximab	8 (27%)	NA	NA
Eternercept	2 (6.7%)	NA	NA
Tocilizumab	2 (6.7%)	NA	NA
Tacrolimus	2 (6.7%)	NA	NA
Salazosulfapyridine	6 (20%)	NA	NA
Bucillamine	5 (17%)	NA	NA
Mizoribine	0 (0%)	NA	NA
Gold	1 (3.3%)	NA	NA

CCP, cyclic citrullinated peptide; CRP, C-reactive protein; DAS28, 28-joint Disease Activity Score; ESR, erythrocyte sedimentaition ratio; HC, healthy control; MMP-3, metalloproteinase-3; NA, not applicable; OA, osteoarthritis; RA, rheumatoid arthritis; SJC, swollen joint count; TJC, tender joint count; VAS, visual analogue scale of general health;

† anti-CCP antibodies of 10 patients were positive among 11 patients examined.

**Table 2:** Clinical features of the participants who contributed synovial fluid

Characteristics	RA	OA
Number of participants	30	30
Sex, male/female	6/24	6/24
Age, mean (range)	63.1 (32 to 88)	75.3 (67 to 89)
Disease duration (y), mean (range)	13.0 (0.5 to 50)	NA
Positive anti-CCP antibody, n (%)	10 (83.3%)^¶^	NA
ESR (mm), mean (range)	49.6 (4 to 116)	NA
CRP (mg/dl), mean (range)	3.1 (0 to 13.9)	NA
MMP3 (ng/ml), mean (range)	362.7 (43.2 to 800)	NA
DAS28, mean (range)	4.9 (2.2 to 7.1)	NA
SJC, mean (range)	4.9 (0 to 17)	NA
TJC, mean (range)	5.1 (0 to 27)	NA
VAS	52.1 (10 to 95)	NA
Steinbrocker Stage, n	I: 3, II: 3, III: 5, IV: 19	NA
Steinbrocker Class, n	I: 1, II: 22, III: 7, IV: 0	NA
Kellgren/Lawrence grade, n	NA	I: 0, II: 0, III: 11, IV: 19
Medication, n (%)		
Prednisolone	20 (67%)	NA
Methotrexate	15 (50%)	NA
Infliximab	3 (10%)	NA
Eternercept	1 (3.3%)	NA
Tocilizumab	0 (0%)	NA
Tacrolimus	2 (6.7%)	NA
Salazosulfapyridine	8 (27%)	NA
Bucillamine	6 (20%)	NA
Mizoribine	1 (3.3%)	NA
Gold	2 (6.7%)	NA

CCP, cyclic citrullinated peptide; CRP, C-reactive protein; DAS28, 28-joint Disease Activity Score; ESR, erythrocyte sedimentaition ratio; MMP-3, metalloproteinase-3; NA, not applicable; OA, osteoarthritis; RA, rheumatoid arthritis; SJC, swollen joint count score; TJC, tender joint count; VAS, visual analogue scale of general health.

¶anti-CCP antibodies of 10 patients were positive among 12 patients examined.

### Preparation for conditioned medium of cells and tissues

PB or joint specimens from RA and OA patients were obtained during joint surgery or from an outpatient clinic. FLSs of RA and OA patients were prepared as previously described [24]. After three to eight passages, FLSs were plated on six-well plates (Corning, NY, USA) in Dulbecco's Modified Eagle's Medium (DMEM; Sigma Aldrich, St. Louis, MO, USA) containing 10% fetal bovine serum (FBS; ICN, Aurora, OH, USA). At confluence, FLSs were washed three times with phosphate-buffered saline (PBS) and cultured in 2 ml of serum-free DMEM for 48 h. Serum-free medium was used to exclude the contamination of miRNAs in bovine serum.

Synovial tissues of 30 mg were incubated at 37°C in 1 ml of serum-free DMEM for 48 h. MNCs from PB and synovial fluid were collected using Histopaque-1077 (Sigma Aldrich) as previously described [24]. One million MNCs were placed on 12-well plates (Corning) and cultured in 1 ml of serum-free RPMI 1640 (Sigma Aldrich) for 48 h. The resultant culture medium was collected, centrifuged 800 g for 10 minutes and stored as conditioned medium at -20°C until analyses.

### RNA isolation

A hundred μl of human plasma or synovial fluid was thawed on ice, diluted with 150 μl of RNase free water and lysed with 750 μl of a phenol-based reagent for liquid sample, Isogen LS (Nippongene, Toyama, Japan). To normalize possible sample-to-sample variation caused by RNA isolation, 25 fmol (total volume of 5 μl) of synthetic *C. elegans *miRNA cel-miR-39 (Hokkaido System Science, Sapporo, Japan), which has no homologous sequences in humans, were added to each denatured sample. Samples were homogenized, incubated for five minutes, added with 0.2 ml chloroform, shaked vigorously for 15 seconds, incubated for three minutes and centrifuged at 12,000 g for 15 minutes at 4°C. Then 300 μl of aqueous phase was applied to High Pure miRNA Isolation Kit (Roche Applied Science, Mannheim, Germany) according to manufacture's protocol.

Total RNA included in 300 μl of conditioned medium was also isolated with High Pure miRNA Isolation Kit according to manufacture's protocol for liquid sample. After samples were mixed with binding buffer, which inhibits RNase activities, 25 fmol of synthetic cel-miR-39 was spiked.

### Reverse transcription and quantitation of miRNAs by real-time PCR

Reverse transcription was performed using NCode VILO miRNA cDNA Synthesis Kit (Invitrogen, Carlsbad, CA, USA) according to the manufacture's protocol. Using EXPRESS SYBR GreenER qPCR SuperMix (Invitrogen), real-time polymerase chain reaction (PCR) was carried out on an Applied BioSystems 7300 Real-Time PCR System (Applied BioSystems, Tokyo, Japan) with standard plasmids generated as in the next paragraph. Forward primers were designed according to NCode miRNA Database [25]. Data were analyzed with SDS Relative Quantification Software version 1.3 (Applied BioSystems, Tokyo, Japan).

Primer sequences were as follows: for hsa-miR-16, 5'-TAG-CAG-CAC-GTA-AAT-ATT-GGC-G-3'; for hsa-miR-132, 5'-TAA-CAG-TCT-ACA-GCC-ATG-GTC-G-3'; for hsa-miR-146a, 5'-TGA-GAA-CTG-AAT-TCC-ATG-GGT-T-3'; for hsa-miR-155, 5'-TTA-ATG-CTA-ATC-GTG-ATA-GGG-GTA-3'; for hsa-miR-223, 5'-TGT-CAG-TTT-GTC-AAA-TAC-CCC-A-3'; for cel-miR-39, 5'-CGT-CAC-CGG-GTG-TAA-ATC-AGC-TTG-3'.

### TA Cloning of PCR products and generation of standard curve

To verify the PCR products and to generate standard curves of miRNAs, thymine adenine (TA) cloning was performed. The resultant reaction buffers of preliminary real-time PCR were directly put in TA cloning using pTAC-1 vector (BioDynamics Laboratory, Tokyo, Japan) according to the manufacture's protocol. We verified that the sequences of inserted approximately 60 nucleotides (about 20 nucleotides of miRNA and about 40 nucleotides added at the reverse transcripts) were all correct, and could not find pre-miRNAs inserted into the vector.

Plasmids with known copy number were put into real-time PCR over an empirically-derived range of copies to generate standard curves for each of the miRNA. Absolute copy number of each target miRNA and spiked cel-miR-39 in samples was obtained according to the generated standard curves. The concentrations of target miRNAs in each sample were calculated according to the obtained absolute copy numbers of spiked cel-miR-39 with known concentration and target miRNAs.

### Statistical analysis

Data were presented as the mean ± standard deviation. Statistical analyses were performed using StatView Ver.5 for Windows (Hulinks, Tokyo, Japan). Differences between two groups were analyzed with Student's *t*-test. Differences among three groups were analyzed with Bonferroni method. Correlations with miRNA concentrations and other clinical factors were analyzed with Pearson product-moment correlation coefficient. The ROCKIT software version 0.9B (Metz, Herman, & Roe, The University of Chicago, Chicago, IL, USA) was used to calculate Receiver Operating Characteristic (ROC) curve values. A *P-*value less than 0.05 was considered statistically significant.

## Results

### The presence and the stability of miRNAs in plasma and synovial fluid

**Figure 1:**
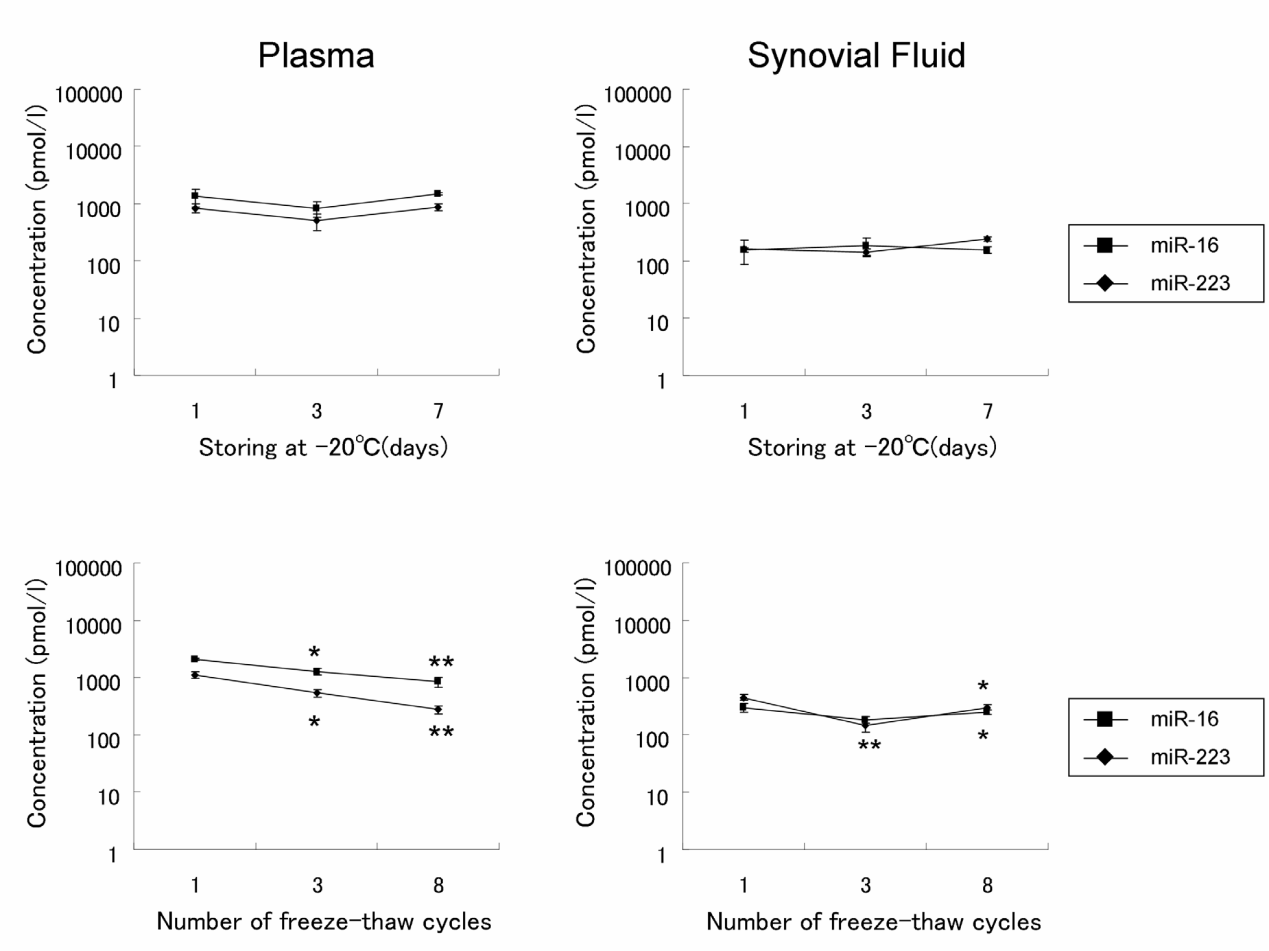
**The presence of miRNAs in plasma and synovial fluid and their stability for storage**. *(Upper) *Plasma and synovial fluid of RA were stored in --20°C until indicated days, thawed and analyzed for the concentrations of the indicated miRNAs. (*Lower*) The concentrations of indicated miRNAs in plasma and synovial fluid of RA after indicated freeze-thaw cycles from --20°C to 4°C. Significant differences compared to the concentration after the first freeze-thaw are indicated by * = *P *< 0.05, ** = *P *< 0.01. Results represent three independent experiments.

It has not been reported whether miRNAs are present in the synovial fluid in a stable form as previously reported in plasma. Both of miR-16 and miR-223 were detectable in both of plasma and synovial fluid (Figure 1). Then, we investigated the stability of plasma and synovial fluid miRNAs for the storage at -20°C and freeze-thaw cycles from -20°C to 4°C. Storage of plasma and synovial fluid at -20°C for up to seven days had minimal effect on concentrations of miR-16 or miR-223 (Figure 1). But concentrations of miRNAs slightly decreased with the number of freeze-thaw cycles (up to eight times), with statistical significances (Figure 1).

### Plasma and synovial fluid miRNAs had distinct profiles

**Figure 2:**
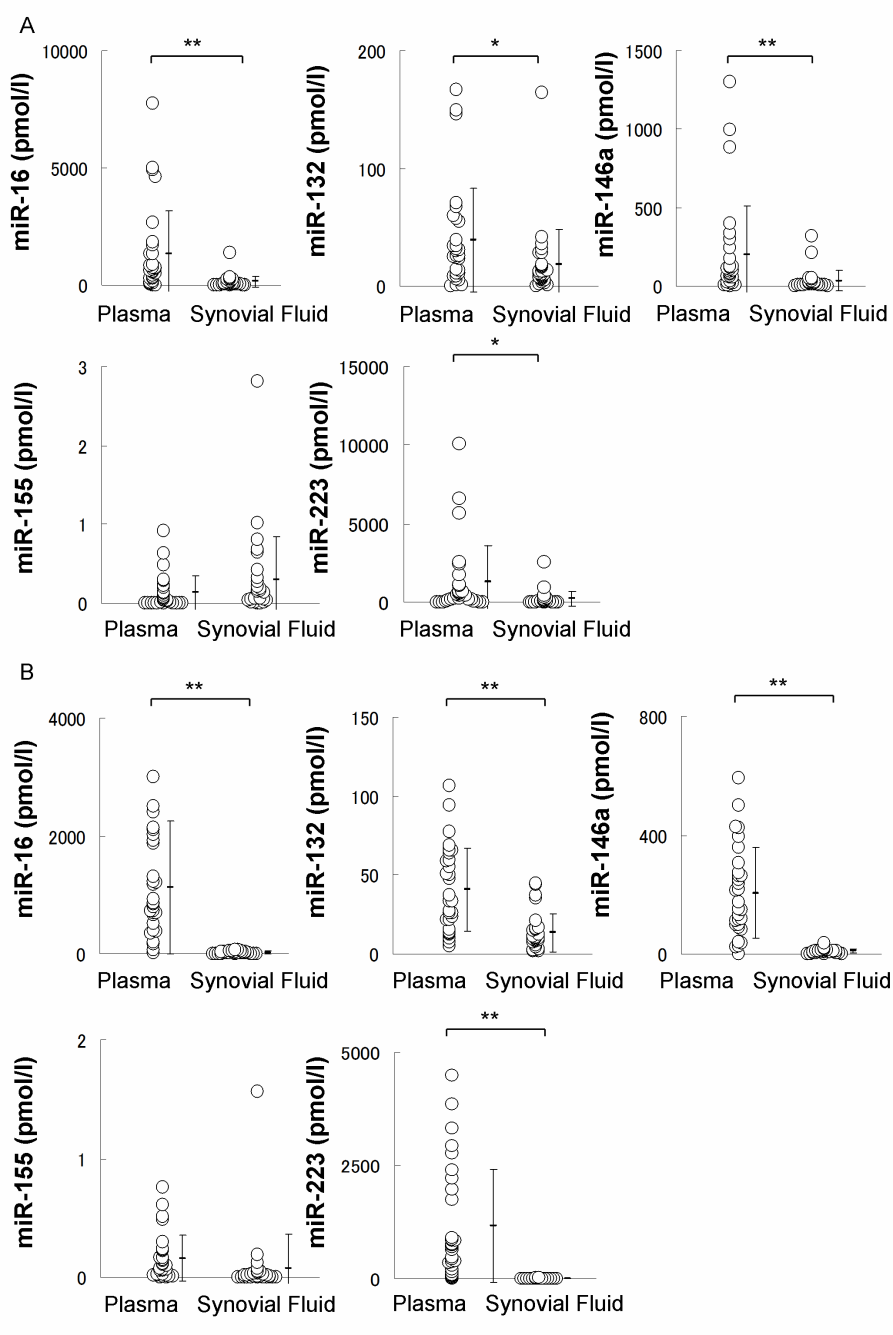
**Comparisons between miRNA concentrations in plasma and those in synovial fluid**. **A and B**, Plasma and synovial fluid concentrations of miR-16, miR-132, miR-146a, miR-155 and miR-223 in RA (A) and OA (B). The average concentrations of these miRNAs were quite different. Significant differences between plasma and synovial fluid are indicated by * = *P *< 0.05, ** = *P *< 0.01.

It is unclear how miRNAs are produced in plasma and synovial fluid. Especially, it is an interesting question whether plasma miRNAs just penetrate into synovial fluid, or tissues facing joint space are generating miRNAs. In RA, the average plasma concentrations of miR-16, miR-132, miR-146a, miR-155 and miR-223 were 1.3*10^3^, 39, 2.0*10^2^, 0.13 and 1.3*10^3 ^pmol/l, respectively, and these in the synovial fluid were 1.5*10^2^, 18, 34, 0.30 and 2.3*10^2 ^pmol/l, respectively. The concentrations of miR-16, miR-132, miR-146a, and miR-223 in synovial fluid were significantly lower than those in plasma (*P *< 0.01, *P *< 0.05, *P *< 0.01 and *P *< 0.05, respectively) (Figure 2A).

In OA, the average plasma concentrations of these miRNAs were 1.1*10^3^, 41, 2.1*10^2^, 0.16 and 1.1*10^3 ^pmol/l, respectively, and these in synovial fluid were 24, 13, 9.3, 7.8*10^-2 ^and 4.6 pmol/l, respectively. The concentrations of miR-16, miR-132, miR-146a and miR-223 in synovial fluid were also significantly lower than those in plasma (*P *< 0.01, *P *< 0.01, *P *< 0.01 and *P *< 0.01, respectively) (Figure 2B).

There were no correlations between plasma miRNA concentrations and synovial fluid miRNA concentrations (Figure S1 in Additional file 1), except miR-223 from OA patients (r = 0.50, *P *= 0.01, n = 22). These findings imply that synovial fluid and plasma miRNAs are distinctly generated.

### Synovial tissues released miRNAs similar to synovial fluid miRNAs

**Figure 3:**
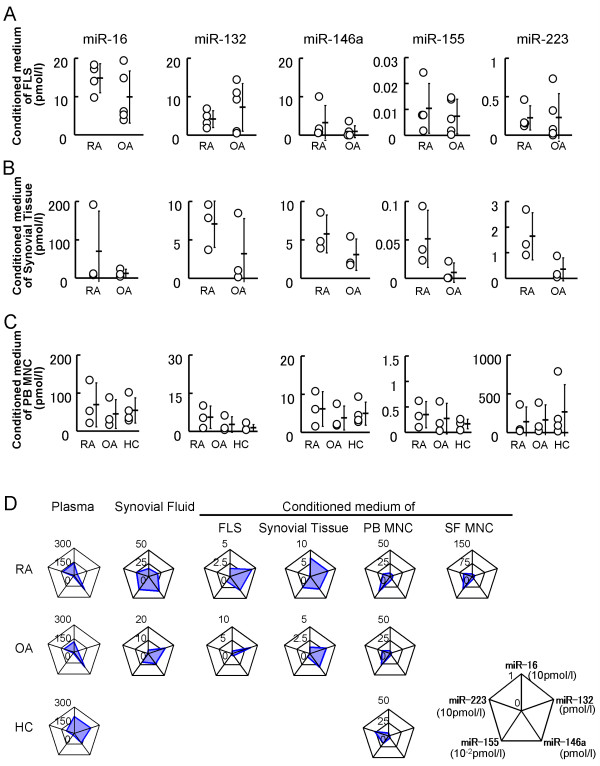
**The concentrations of miRNAs in the condition medium of each cell or tissue of RA and OA**. **A**, FLSs of RA (n = 4) and OA (n = 5) were cultured in serum-free medium for 48 h. Concentrations of miRNAs in each conditioned medium are shown. **B**, Synovial tissues of RA (n = 3) and OA (n = 3) were cultured in serum-free medium for 48 h. Concentrations of miRNAs in conditioned medium are shown. There were no statistically significant differences between RA and OA in A and B. **C**, PB MNCs of RA (n = 3), OA (n = 3) and HC (n = 3) were cultured in serum-free medium for 48 h. Concentrations of each miRNA in conditioned medium are shown. There were no statistically significant differences among RA, OA and HC. **D**, Radar charts show the average concentrations of each miRNA of each sample. Expression patterns of plasma miRNA of RA and OA were similar. Synovial fluid miRNAs were similar to the miRNAs secreted by synovial tissues.

To estimate the origin of plasma or synovial fluid miRNAs, FLSs, synovial tissues, PB MNCs, and synovial fluid MNCs were cultured with serum-free medium for 48 h, and miRNAs in the resultant conditioned medium were measured (Figure 3A, B, C). There were no statistically significant differences in analyzed miRNAs between RA and OA. However, radar charts of the mean concentration of each miRNA showed the difference in secretion patterns of miRNAs between tissues (Figure 3D). FLSs and synovial tissues secreted miR-132 with relatively high concentration, but rarely miR-223, while MNCs secreted miR-223 and miR-155 moderately, but miR-16, miR-132 and miR-146a at relatively low level. Plasma miRNAs seemed to originate not limited just MNCs because miR-146a in plasma was relatively higher than that secreted by MNCs. Rader charts also indicated that synovial fluid miRNAs and plasma miRNAs have different origins because of the different patterns in miR-132 and miR-155 (Figure 3D). Interestingly, synovial fluid miRNAs of OA were most similar to miRNAs secreted by synovial tissues. In RA, the expression pattern of miR-16, miR-132, miR-146a, and miR-155 of synovial fluid was similar to that secreted by synovial tissues, while synovial fluid miR-223 was relatively high compared to miR-223 secreted by synovial tissues. Synovial tissues appear a main source of synovial fluid miRNAs, but synovial fluid miR-223 reflects the influence of cells including MNCs infiltrating into synovial fluid. These results indicate that synovial fluid miRNAs reflect the condition of joint space.

### Plasma miRNAs differentiated RA and OA from HC

**Figure 4:**
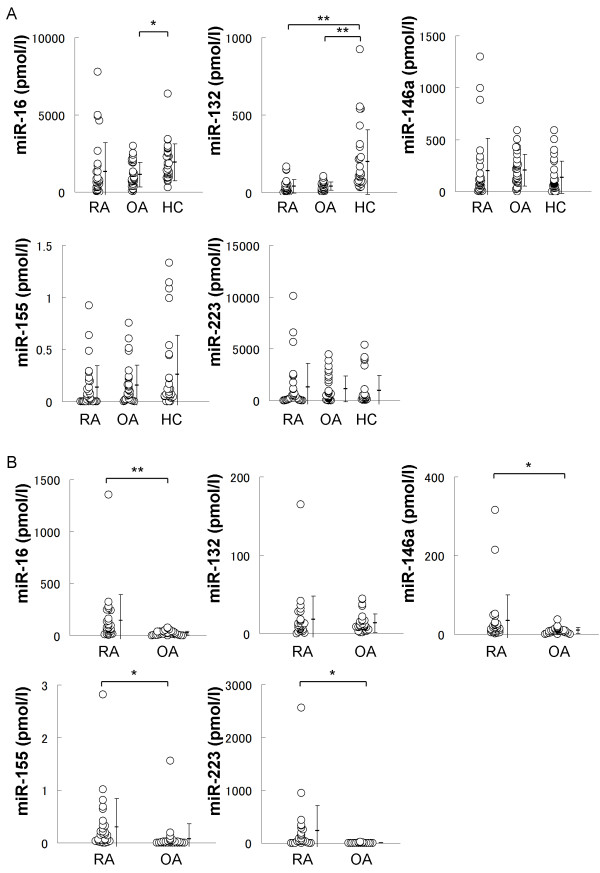
**The concentrations of plasma and synovial fluid miRNAs in RA and OA**. **A**. Plasma concentrations of miR-16, miR-132, miR-146a, miR-155 and miR-223 in RA, OA and HC. The plasma concentration of miR-16 in OA was significantly lower than HC. The plasma concentrations of miR-132 in RA and OA were significantly lower than HC. **B**. Synovial fluid concentrations of indicated miRNAs in RA and OA. The concentrations of miR-16, miR-146a miR-155, miR-223 in RA were significantly higher than those in OA. Significant differences are indicated by * = *P *< 0.05, ** = *P *< 0.01.

Plasma miRNAs have been expected as biomarkers of malignant tumors [14,17]. To determine whether plasma miRNAs can be clinical markers for RA or OA, plasma samples from RA, OA patients and HC were analyzed (Figure 4A). As suggested in radar charts (Figure 3D), plasma miR-132 of patients with RA or OA was lower than that of HC with statistical significances (*P *< 0.01 or *P *< 0.01). Plasma miR-16 of patients with OA was lower than that of HCs with statistical significance (*P *< 0.05). Thus, investigated plasma miRNAs of RA and those of OA were somehow similar, but significantly different from those of HC.

### Plasma miR-132 can be a potential diagnostic marker for patients with RA and OA

**Figure 5:**
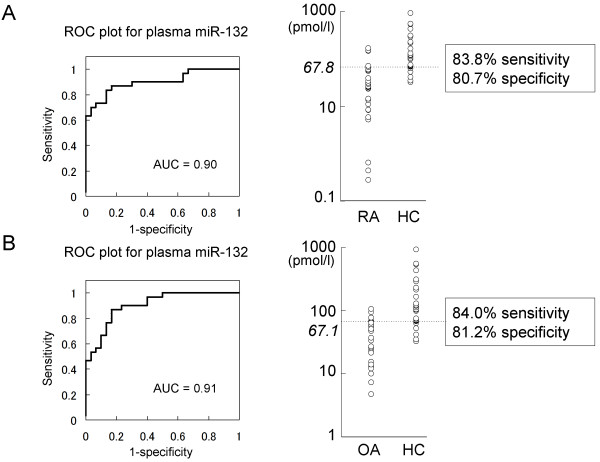
**ROC curve analysis of plasma miR-132 to differentiate patients with RA or OA from HCs**. **A**. ROC plot of plasma miR-132 for the diagnosis of RA. AUC was 0.90. A cutoff value of 67.8 pmol/l diagnosed RA at the sensitivity of 83.8% and the specificity of 80.7%. **B**. ROC plot of plasma miR-132 for the diagnosis of OA. AUC was 0.91. A cutoff value of 67.1 pmol/l diagnosed OA at the sensitivity of 84.0% and the specificity of 81.2%.

To determine the diagnosability of plasma miR-132 for patients with RA or OA, we conducted a ROC analysis of miR-132 (Figure 5A, B). Plasma miR-132 test at a cutoff value of 67.8 pmol/l could detect individuals with RA at 83.8% of sensitivity and 80.7% of specificity, and plasma miR-132 test at a cutoff value of 67.1 pmol/l could also detect individuals with OA at 84.0% of sensitivity and 81.2% of specificity. Area under the ROC curve (AUC) of each plot was not lower than 0.90, indicating high diagnosability of each test.

### Synovial fluid miRNAs differentiated RA and OA

While analyzed plasma miRNAs failed to differentiate RA and OA, synovial fluid miRNAs had a possibly to differentiate them because synovial fluid miRNAs reflected the condition of joint space more than plasma miRNAs (Figure 3D). Synovial fluid miR-16, miR-146a miR-155 and miR-223 of patients with RA were higher than those of patients with OA with statistical significances (*P *< 0.01, *P *< 0.05, *P *< 0.05 and *P *< 0.05, respectively) (Figure 4B). Additionally we compared ratio of concentration of each synovial fluid miRNA to plasma miRNA (SF/PB ratio) between RA and OA (Figure S2 in Additional file 2). Similar to the result of synovial fluid miRNAs, SF/PB ratios of miR-16, miR-146a miR-155 and miR-223 were significantly higher in RA than those in OA (*P *< 0.05, *P *< 0.05, *P *< 0.01, *P *< 0.01, respectively). These results indicate that synovial fluid miRNAs can be a useful tool for diagnosis of RA and OA, and for the analysis of their pathogenesis.

### Plasma miRNAs and synovial fluid miRNAs correlate with clinical variables

**Figure 6:**
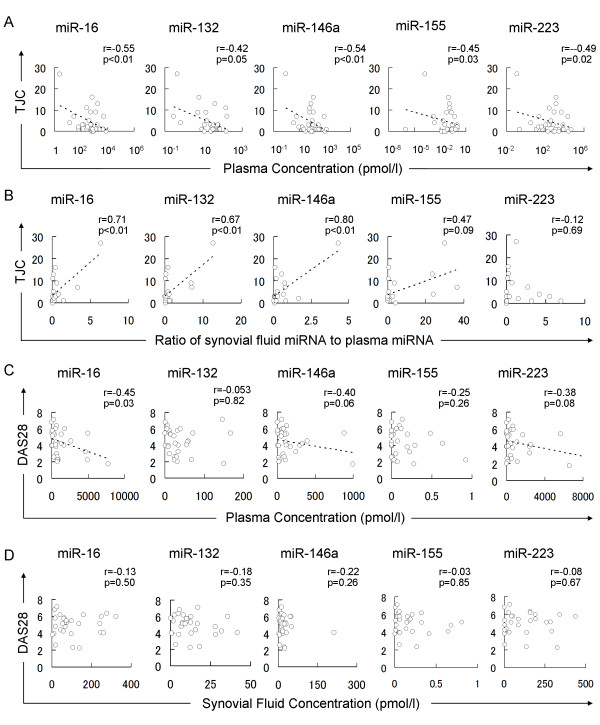
**Correlation between disease activities of RA and miRNAs in plasma or synovial fluid**. TJC correlated with plasma miRNAs **(A) **or SF/PB ratio of miRNA **(B)**. DAS28 correlated with plasma miRNAs **(C)**, but not with synovial fluid miRNAs **(D)**. r values of Pearson's product-moment correlation and *P*-values of their null hypothesis are shown. Regression lines are shown when *P *values are less than 0.1.

To assess the possibility of plasma and synovial fluid miRNAs as biomarkers of RA, we investigated the correlation of miRNAs with clinical variables including serum matrix metalloproteinase-3 (MMP-3), C-reactive protein (CRP), Erythrocyte Sedimentation Rate (ESR), 28-joint Disease Activity Score (DAS28), swollen joint count (SJC) and tender joint count (TJC). Although plasma miRNAs did not significantly correlate with MMP-3, CRP, or ESR, plasma miR-16, miR-146a, miR-155, and miR-223 inversely correlated with TJC (r = - 0.55, *P *< 0.01, n = 30; r = - 0.54, *P *< 0.01 n = 30; r = - 0.45, *P *= 0.03, n = 30; r = - 0.49, *P *= 0.02, n = 30; respectively) (Figure. 6A), and plasma miR-16 also inversely correlated with DAS28 (r = - 0.45, *P *= 0.03, n = 30) (Figure 6C). Unexpectedly, synovial fluid miRNAs had no correlations with clinical variables of RA including DAS28 (Figure 6D). Then, we hypothesized the relative expression of synovial fluid miRNAs compared to plasma miRNAs would more reflect the condition of joint of RA than absolute concentration of synovial fluid miRNAs. Dot plots of TJC and SF/PB ratio are shown in Figure 6B. Although SF/PB ratio of each miRNA failed to correlate with DAS28 (data not shown), SF/PB ratio of miR-16, miR-132 and miR-146a correlated with TJC (r = 0.71, *P *< 0.01, n = 20; r = 0.67, *P *< 0.01, n = 20; and r = 0.80, *P *< 0.01, n = 20) at higher R^2 ^values than plasma miRNAs.

## Discussion

Tissue miRNAs have been noted not only as key molecules in intracellular regulatory networks for gene expression, but also as biomarkers for various pathological conditions [26]. Recent studies suggest that miRNAs in plasma can be biomarkers for the diagnosis of lung, colorectal and prostate cancer [14,27]. Plasma miRNAs are also suggested to be potential biomarkers for drug-induced liver injury, and myocardial injury [28,29]. In this report, we showed the presence and the stability of miRNAs in synovial fluid and plasma. We also found that the expression of miRNAs in synovial fluid was distinct from that in plasma and may reflect the condition of joint space. Consistently, synovial fluid concentrations of miR-16, miR-146a, miR-155 and miR-223 were significantly higher in RA than those in OA. Finally we referred the possibility of plasma and synovial fluid miRNAs as potential biomarkers of RA.

We quantified miRNAs by real-time PCR after using NCode VILO miRNA cDNA Synthesis Kit. This kit polyadenylates miRNAs and reverse-transcribes with a poly(T) adapter as reverse primer. Because the specificity of this procedure depends on the annealing of the forward primer to the sequence of mature miRNA in the amplicon during amplification, there is a low possibility that pre-miRNAs are also amplified [30]. To exclude the contamination of pre-miRNAs and nonspecific amplification, we performed TA cloning of PCR products. We verified that all the inserted size was approximately 60 nucleotides by electrophoresis, and that sequences were correct. These results were probably attributed to low abundance of pre-miRNAs and difficulties in polyadenylation of pre-miRNA due to the presence of the stem loop structure [31]. Even if there remains little possibility to amplify pre-miRNA, we think that procedures used in this study are useful for diagnosis and determination of activities.

Plasma miRNAs have been shown to be remarkably stable in plasma and protected from endogenous RNase activity [14]. In previous reports, plasma miRNAs are stable at room temperature for up to 24 h and resistant for freeze-thawing from -80°C to room temperature up to eight times. We additionally demonstrated that miRNAs in synovial fluid were as stable as miRNAs in plasma and that both of these miRNAs were also stable at -20°C for up to seven days. These stabilities contribute to the handiness of plasma and synovial fluid miRNAs as biomarkers.

Although we showed that synovial tissue is a main source of synovial fluid miRNA, the mechanism for stability of synovial fluid miRNA remains to be determined. In plasma, some miRNAs are thought to be secreted in a form of exosomes, which are 50- to 90-nm membrane vesicles abundant in plasma containing mRNAs and miRNAs [32-34]. Exosomes released from various cells can transfer proteins and RNA between cells, facilitating processes such as antigen presentation and in trans signaling to neighboring cells [34-37]. However, other mechanisms for stabilization may exist (for example, in a RNA-induced silencing complex (RISC)), because some miRNAs were reported to be biomarkers of tissue injury (for example, liver, heart, kidney, *et al*.). Exosomes were shown to exist in synovial fluid [38], but there have been no report about the existence of miRNA in synovial fluid or its exosomes.

Investigated miRNAs in this study have already been shown to associate with RA or OA. miR-16 and miR-132 were shown to be upregulated in PB MNCs of RA patients [21]. Although the function of miR-16 and miR-132 in RA has not been determined yet, miR-16 is present in high levels in most of cells and thought to be potentially a *master miRNA *involved in determining mRNA stability via AU-rich element sites [39]. miR-146a is upregulated in PB MNCs, FLS and synovial tissue of RA [19-21] and expressed in cartilage of low-grade OA [40]. The targets of miR-146a/b are IL-1β and TRAF6, which is a key molecule in the down stream of TNFα and IL-1β signaling [41]. The expression of miR-155 is upregulated in RA FLS and has repressive effect to MMP-3 and 1 [18]. The expression of miR-223 is down regulated in RA FLS [19].

Our hypothesis was that in RA patients, miR-16, miR-132, miR-146 and miR-155 were upregulated in plasma and synovial fluid, but miR-223 down regulated. However, there were no statistically significant differences between plasma miRNAs of RA and those of OA. These results are not inconsistent with the previous report: Expression patterns of exosomal miRNAs were shown to be different from those of intracellular miRNAs [34], though we could not directly show that the synovial fluid miRNA exist in the form of exosome. We showed synovial fluid miRNAs were similar to miRNAs secreted by synovial tissues, while plasma miRNAs were different from miRNAs secreted by MNCs. These facts suggest that synovial tissues and infiltrating cells are a main source of synovial fluid miRNAs, while plasma miRNAs are generated by various tissues.

In this study, all healthy controls were younger than 66 years old according to the request of our ethical committee, while patients with OA were older than 64 years old. When the age of patients and healthy controls was limited from 40 to 60 years to match the age background of groups, plasma miR-132 of HC (n = 9) was still significantly higher than that of RA (n = 16) (*P *< 0.01). This result suggests that the difference in age between groups has little effect on our analyses.

Plasma concentration of miR-132 differentiated patients with RA or OA from HC, though plasma and synovial fluid miR-132 failed to differentiate RA from OA. Furthermore, plasma miR-132 or its SF/PB ratio correlated with TJC. These results indicate that miR-132 might be involved in the systematic condition of patients with joint inflammation.

On the other hand, miR-16, miR-146a, miR-155 and miR-223 were higher in RA synovial fluids than in OA synovial fluids. Although these miRNAs of plasma had no differences between RA and OA, they significantly correlated with TJC, and plasma miR-16 also correlated with DAS28. Moreover, SF/PB ratio of miR-16 and miR-146a also correlated with TJC with moderate R^2 ^values. These collectively imply that miR-16, miR-146a miR-155 and miR-223 are involved in the pathogenesis specific for RA.

As reported in the field of malignant tumors [14,16,17], disease specific plasma miRNAs for RA or OA are expected. Although investigated plasma miRNAs failed to differentiate RA and OA, disease specific miRNAs that are not investigated in this study may exist. In our preliminary study, miR-124a, miR-142-3p, miR-142-5p, and miR-133a were also detectable. Further analysis for comprehensive plasma and synovial fluid miRNAs using larger number of samples including age-matched RA and OA patients with various severity and healthy controls are expected.

## Conclusions

In this study, we have firstly shown that miRNAs are present and stable in synovial fluid. Synovial fluid miRNAs showed distinct profiles from plasma miRNAs, implying generated chiefly from synovial tissues, and clearly differentiated RA and OA. Synovial fluid and plasma miRNAs can be promising diagnostic biomarkers and potential sources for analyzing roles of miRNAs in RA and OA.

## Abbreviations

AUC: areas under the ROC curve; CRP: C-reactive protein; DAS28: 28-joint Disease Activity Score; DMEM: Dulbecco's Modified Eagle's Medium; ESR: erythrocyte sedimentation rate; FLS: fibroblast-like synoviocyte: HC: healthy control; miRNA: microRNA; MMP-3: matrix metalloproteinase-3; MNC: mononuclear cell; OA: osteoarthritis; PB: peripheral blood; PCR: polymerase chain reaction; PBS: phosphate-buffered saline; RA: rheumatoid arthritis; RISC: RNA-induced silencing complex; ROC: Receiver Operating Characteristic; SF/PB ratio: ratio of concentration of synovial fluid miRNA to plasma miRNA; SJC: swollen joint count; TJC: tender joint count; TA: thymine adenine.

## Competing interests

H Yoshitomi and K Murata are applying for a patent relating to the content of the manuscript. The authors do not receive any reimbursements, fees, funding, or salary from an organization that holds or has applied for patents relating to the content of the manuscript. The other authors declare that they have no competing interests.

## Authors' contributions

KM conducted all experiments and drafted the manuscript. HY designed the experiment, recruited study subjects, assisted with statistical evaluation, and edited the manuscript. ST, MI and KN collected patients' samples. HI and TN recruited study subjects, provided clinical insights and advice. All authors read and approved the final manuscript.

## Supplementary Material

Additional file 1 **Supplementary Figure S1**. Correlation between plasma miRNA and synovial fluid miRNA. There were no correlations between plasma miRNA concentrations and synovial fluid miRNA concentrations of patients with RA **(A) **or OA **(B)**, except miR-223 from OA patients. Click here for file

Additional file 2 **Supplementary Figure S2**. Comparison of SF/PB ratio of miRNA between RA and OA. Significant differences between RA and OA are indicated by * = *P *< 0.05, ** = *P *< 0.01. Click here for file
