# Supplementary figures and images for: Plasma and synovial fluid microRNAs as potential biomarkers of rheumatoid arthritis and osteoarthritis
Source: Arthritis Res Ther. 2010 May 14;12(3):R86. doi: 10.1186/ar3013 (PMC2911870; doi:10.1186/ar3013)

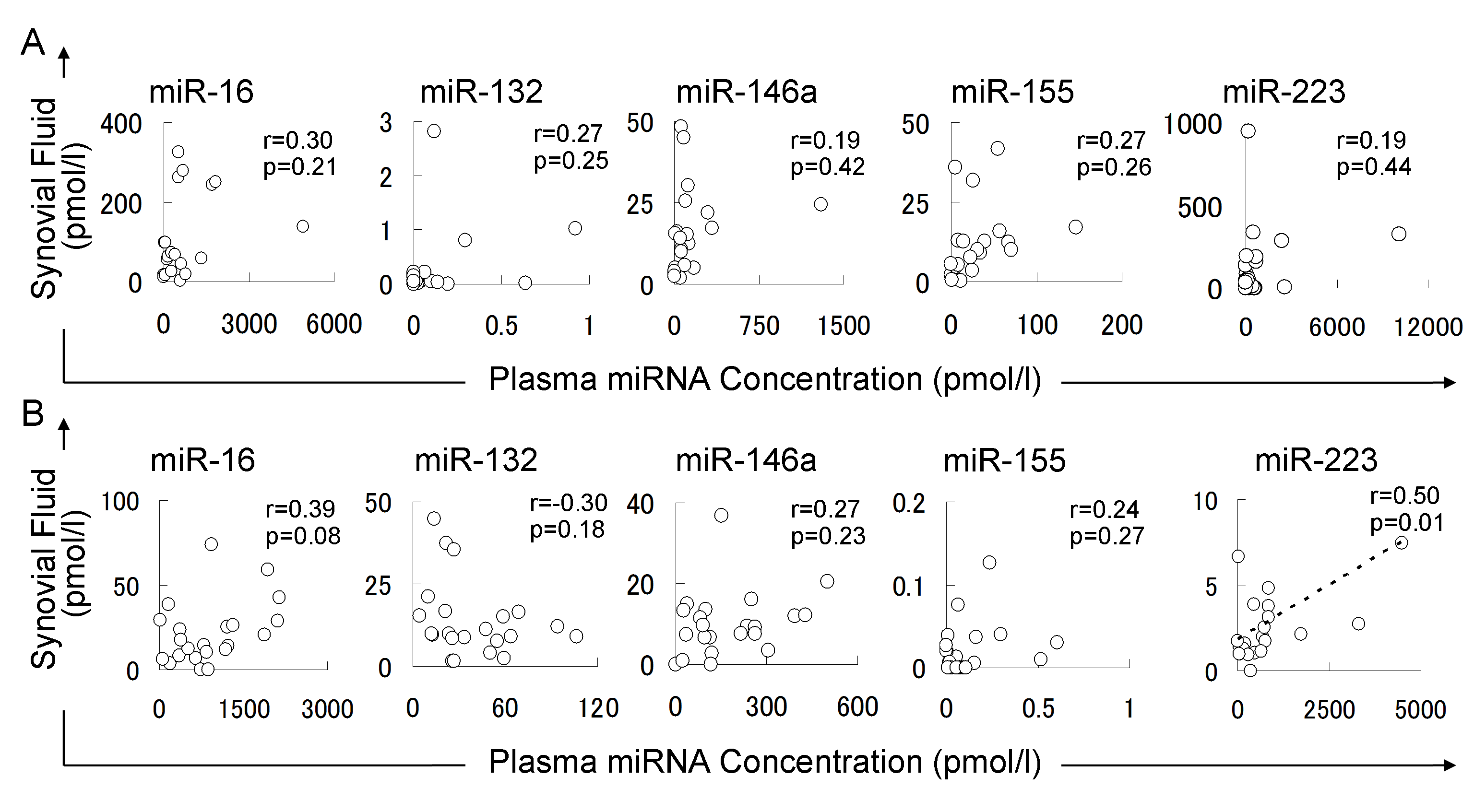

Supplement: Additional file 1 — Supplementary Figure S1. Correlation between plasma miRNA and synovial fluid miRNA. There were no correlations between plasma miRNA concentrations and synovial fluid miRNA concentrations of patients with RA (A) or OA (B), except miR-223 from OA patients. [file ar3013-S1.TIFF]

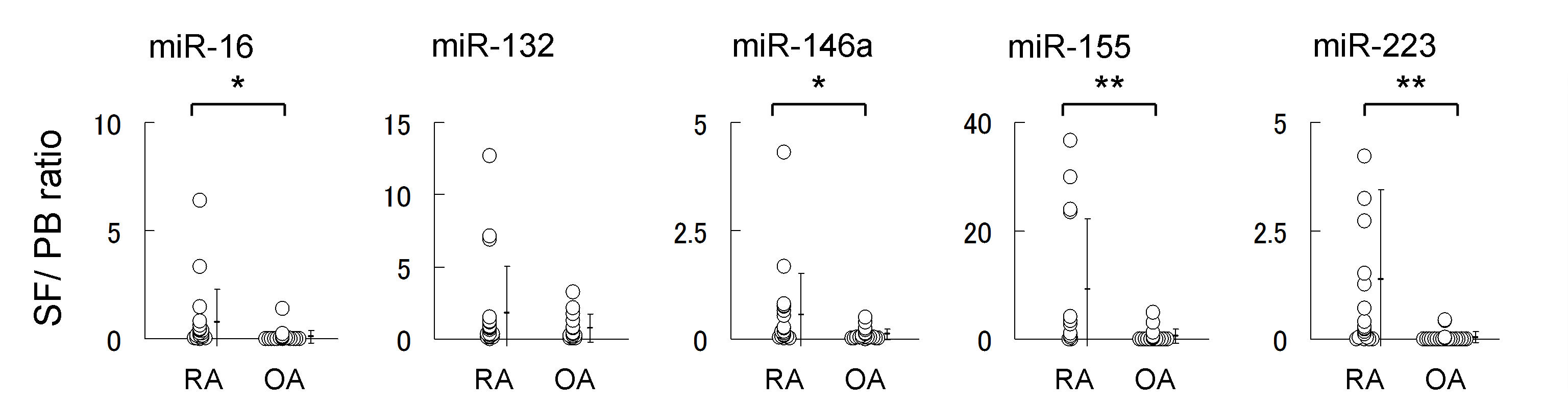

Supplement: Additional file 2 — Supplementary Figure S2. Comparison of SF/PB ratio of miRNA between RA and OA. Significant differences between RA and OA are indicated by * = P < 0.05, ** = P < 0.01. [file ar3013-S2.TIFF]
